# Cytomorphometric Changes in Hippocampal CA1 Neurons Exposed to Simulated Microgravity Using Rats as Model

**DOI:** 10.3389/fneur.2014.00077

**Published:** 2014-05-20

**Authors:** Amit Ranjan, Jitendra Behari, Birendra N. Mallick

**Affiliations:** ^1^School of Life Sciences, Jawaharlal Nehru University, New Delhi, India; ^2^School of Environmental Sciences, Jawaharlal Nehru University, New Delhi, India

**Keywords:** dendritic arborization, Golgi–Cox, learning and memory, length of active zone, neuronal plasticity, spine density

## Abstract

Microgravity and sleep loss lead to cognitive and learning deficits. These behavioral alterations are likely to be associated with cytomorphological changes and loss of neurons. To understand the phenomenon, we exposed rats (225–275 g) to 14 days simulated microgravity (SMg) and compared its effects on CA1 hippocampal neuronal plasticity, with that of normal cage control rats. We observed that the mean area, perimeter, synaptic cleft, and length of active zone of CA1 hippocampal neurons significantly decreased while dendritic arborization and number of spines significantly increased in SMg group as compared with controls. The mean thickness of the postsynaptic density and total dendritic length remained unaltered. The changes may be a compensatory effect induced by exposure to microgravity; however, the effects may be transient or permanent, which need further study. These findings may be useful for designing effective prevention for those, including the astronauts, exposed to microgravity. Further, subject to confirmation, we propose that SMg exposure might be useful for recovery of stroke patients.

## Introduction

Human astronauts who spent more than 400 days in space showed ataxia, perceptual illusions, neuromuscular weakness, and fatigue after landing. They tended to readapt with time under normal gravity condition after returning to the earth (1). During space travel, astronauts are exposed to many factors including microgravity and hypergravity. Exposure to microgravity induces changes in the brain, which possibly are underlying causes that are reflected as many of the altered physio-behavioral changes seen in the astronauts. Independent studies have reported that exposure to microgravity alters levels of biomolecules (2–4), increase cortical spine density (5), induce impaired cognition as well as learning and memory (6, 7). These impairments are likely to be specific to exposure to microgravity because such microgravity exposure-associated loss of memory (8, 9) tended to reverse with time after their return to the earth under normal gravity condition (10). Notwithstanding, other studies have reported that 6 (11) and 8 (12, 13) days spaceflight showed no impairment of memory, however, 13 (14) and 16 (10) days exposure showed some impairment. Therefore, microgravity exposure-associated impairments of cognitive functions are likely to be dependent on duration of exposure and the effects varied depending on the rate of recovery and degree of adaptation. On the other hand, isolated studies have shown that impaired learning and memory is associated with reduced spine density (15) and neuronal loss (16, 17).

Apparently there is an inherent contradiction that exposure to microgravity caused impairment of memory associated with increased spine density, while otherwise memory impairment is associated with neuronal loss and reduced spine density, which indeed needed investigation under suitable controlled experimental (simulated) conditions. One possibility could be that the changes in spine density due to exposure to microgravity could be a compensatory effect and there could be other micro-cytomorphological changes as an indication of initial signs of possible loss of neurons (18, 19), which were unknown. Further, as hippocampal neurons are involved in memory formation (20) and the latter is altered by exposure to microgravity (6, 8), we argued that exposure to simulated microgravity (SMg) under controlled experimental condition is likely to affect the hippocampal CA1 neurons. Therefore, in this study, we exposed rats to 14 days SMg and evaluated CA1 neuronal cytomorphometry, arborization, dendritic spine density, synaptic cleft (SC), length of active zone (LAZ), and thickness of postsynaptic density (PSD). It was observed that neuronal morphology and synaptic connectivity were damaged suggesting initial changes of neuronal loss; however, the silver lining is that there is a possibility of compensation and recovery.

## Materials and Methods

### Animal preparation

**Figure 1:**
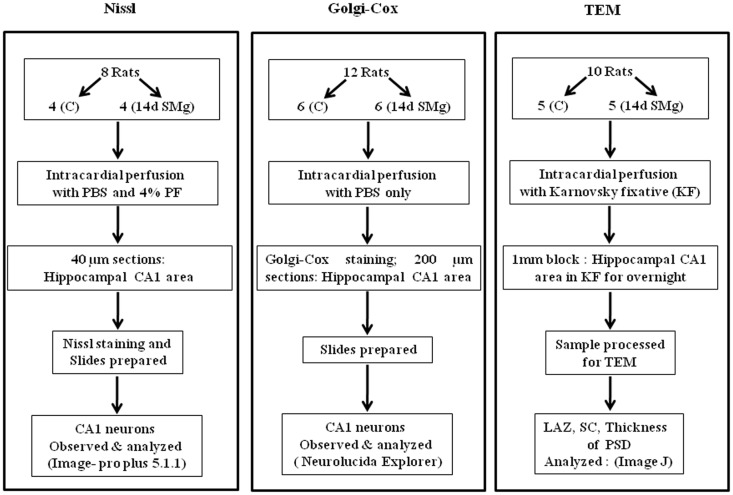
**Flow chart of the methods used for sample preparations in this study**. Abbreviations: as in text.

Experiments were conducted on inbred male Wistar rats (225–275 g) maintained in 12/12 h light/dark cycle with food and water *ad libitum*. NIH guidelines were followed while conducting experiments and all experiments were approved by the Institutional Animal Ethics Committee of Jawaharlal Nehru University. Every step was taken to reduce number of animals used and their sufferings. Rats were subjected to SMg using hind-limb suspended (HLS) model, which has been widely used for such studies (21–23), while control rats were maintained in their normal cages in the same room to rule out non-specific effects. For SMg exposure, the rats were lifted with a tail harness that raised their hind-limbs 1 cm off the cage floor in a 30° head-down angle and maintained for 14 days. We have considered only those experimental rats for cytomorphometric analysis in which the body weight of experimental rats followed the pattern as reported earlier (22). Commenting on the weight of normal adult rats (>200 g), upon exposure to SMg it has been stated that “*rats may lose or gain weight more slowly for several days after initial unloading, followed by stabilization of body weight*” (22). In this study, we observed that weight loss or gain was not consistent up to the seventh day of exposure. However, thereafter the rats gained weight, which was stabilized by the 14th day of exposure and hence 14-day exposure paradigm was used in this study. The animals were randomly divided into three groups (Figure 1).

### Nissl-staining method

At the end of experiment, eight rats (four control and four experimental) were anesthetized with ketamine–xylazine (80 and 32 mg/kg, respectively, i.p.; Chandra Bhagat Pharma Pvt. Ltd., India). The brains were intracardially perfused with 0.1 M phosphate buffer saline (PBS) and 4% paraformaldehyde in 0.1 M phosphate buffer (PB) at pH 7.4. Brains were fixed overnight in the same fixative and were cryoprotected in 30% sucrose in PBS. Thereafter, 40 μm frozen sections (Leica, Solms, Germany) through the antero-posterior extension of hippocampus [between bregma −2.30 and −5.30 mm as in atlas of Paxinos and Watson (24)] were taken onto gelatin-coated slides, processed for Nissl-staining, and analyzed as reported earlier in detail (25, 26). Briefly, the sections were stained with 1% cresyl violet and 0.1% thionin in acetate buffer following standard protocol of Nissl-staining. The stained sections were dehydrated in different grades of ethanol, cleared in xylene, cover slipped with distrene plasticizer xylene (DPX), and air dried before viewing under microscope for cytomorphometric analysis. The slides were coded before the CA1 hippocampal neurons were analyzed. The boundaries of 30–40 neuronal perikarya from every third serial section were traced on the computer screen (with the help of a computer mouse) and their areas as well as perimeters estimated by using cytomorphometric analysis software (Image-Pro plus 5.1.1). Six to eight such sections from each animal were estimated and there were four animals per treatment group. Thus, on an average 850 ± 50 neurons were estimated from anatomical CA1 brain areas of experimental as well as control rats. The mean area and perimeter data were used to calculate the soma form factor (FF) (4π area/perimeter^2^), which is a parameter to comment on shape of the cell. The cells having FF values closure to 1 would be relatively rounder in shape than those closure to 0 (27). Further, rough surfaced cells would have greater possibility to change FF than smoother surfaced cells (25, 26).

### Golgi–Cox impregnation method

In Nissl-staining, the neurons were seen in one plane (2-D). However, as neurons occupy volume in space, to get information in 3-D, we used Golgi–Cox impregnation method and reasonably extrapolated possible changes in CA1 neurons after exposure to 14 days SMg. Brains of another set of six each of control and experimental rats were treated with Golgi stain as reported earlier (28). Anesthetized rats were perfused transcardially with 0.1 M PBS solution at pH 7.4. All the brains were removed, washed with distilled water followed by freshly prepared Golgi–Cox solution (29). The freshly prepared Golgi–Cox solution contained 5 parts of 5% potassium dichromate, 5 parts of 5% mercuric chloride, 4 parts of 5% potassium chromate in 10 parts of double distilled water. The brains were placed in a brain slicer (WPI, USA) and 5 mm thick coronal blocks containing the antero-posterior extension of hippocampus were taken out from each rat brain. Each tissue block was placed in separate cotton-lined dark colored glass bottle containing 25–30 ml Golgi–Cox solution at 37°C for 72 h and slides were prepared as reported earlier (28, 30). In brief, 200 μm thick coronal sections through hippocampus were cut using a vibratome (3000 series, Evergreen Blvd., St. Louis, MO, USA). The sections were rinsed, dehydrated with 50% of ethanol, and transferred into ammonia solution (3:1, 25% ammonia: distilled water) for staining. The stained sections were rinsed, treated with 5% sodium thiosulfate in dark, rinsed in distilled water, dehydrated through increasing grades of alcohol (70, 80, 95% of ethanol and 99% of 1-butanol), cleared in toluene, and mounted in DPX on gelatin-coated slides. The slides were allowed to dry at room temperature for 72 h before observing under microscope for cytomorphological analysis. The control and experimental slides were coded and randomized to minimize bias.

The Golgi-impregnated pyramidal CA1 neurons from the hippocampus were readily identified by their characteristic triangular soma shape, apical dendritic extension toward the center, and many dendritic spines. The following criteria were used to select pyramidal neurons for analysis and reconstruction: (1) localization of a neuron in the CA1 area; (2) presence of untruncated dendrites; (3) full impregnation of the neurons; (4) relative isolation from neighboring impregnated neurons to avoid interference while analyzing. The spines were identified and counted based on the morphological criteria. Only protrusions perpendicular to the dendritic shaft that possessed a clear neck and bulbous head were counted.

To analyze effects of 14 days SMg on CA1 neurons, we carried out cytomorphological analysis of five to six neurons per animal in four to six sections. Thus, 28 ± 2 neurons in each experimental and control rats were traced (1000×) and analyzed using a digital camera (MBF CX9000) attached to the microscope (Olympus BX51, Japan) using dedicated software Neurolucida 9 (MBF Biosciences, USA).

### Transmission electron microscope method

In separate sets, transmission electron microscope (TEM) was used to evaluate the changes in SC, thickness of post PSD and LAZ of CA1 neurons from five control and five experimental rats. Anesthetized rats were intracardially perfused with 200 ml of Karnovsky fixative. The brains were taken out, trimmed from all sides of expected hippocampal CA1 area to obtain approximately 1–1.5 mm^3^ pieces. All such samples containing the CA1 area were fixed overnight at 4°C in Karnovsky’s fixative. Those tissues were then processed for TEM in Advanced Instrumentation Research Facility of our university. Samples were washed in 0.1 M PB followed by post fixation in 1% osmium tetroxide in 0.1 M PB. The samples were then washed, dehydrated in the ascending grades of acetone, cleared in toluene followed by infiltration, and embedded in araldite mixture (CY 212). The samples were labeled so that the analysis could be done unbiased to analyzer. One micrometer sections were cut using ultra microtome (Leica EMUC6, Austria) and stained with toluidine blue to identify the orientation of neurons. Ultra-thin sections (80 nm) were picked on meshed copper grids, and stained with uranyl acetate and lead citrate. The sections were viewed using microscope (TEM JEOL 2100F, Japan) and 10–12 images of Gray’s type-1 asymmetric synapse (magnification 30000×) from each rat brain were captured at random for analysis. Synapses were identified by the presence of presynaptic and postsynaptic membrane thickening and the presence of synaptic vesicles in the presynaptic (terminal) zone. The width of the SC (mean of distances at three to four sites including the widest, intermediate, and least), the thickness of PSD at thickest part, and the LAZ were measured as reported earlier (31) using software Image J. Thus, on an average 60 ± 5 synapses were analyzed from five each of control and experimental rats.

### Statistical analysis

The data was expressed as mean ± SEM. Data from experimental and control groups were statistically analyzed applying one-way analysis of variance (ANOVA) followed by Holm–Sidak test and *P* ≤ 0.05 was considered significant using Sigma Stat software (Jandel Scientific, USA).

## Results

### Nissl-staining

**Figure 2:**
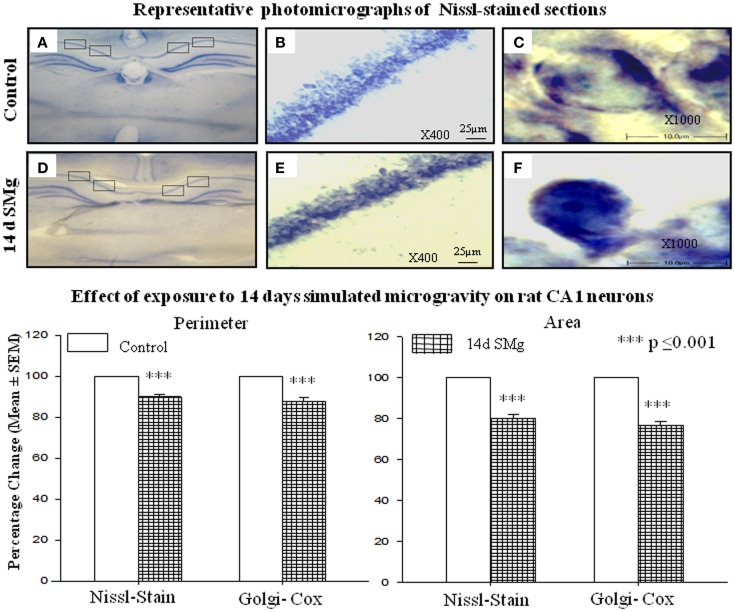
**Representative photomicrographs of Nissl-stained sections under control and 14 days SMg conditions are shown in this figure**. **(A,D)** Representative complete sections having four blocks in each section. Neurons in each block were analyzed as described in the text. **(B,E)** 400× magnifications and CA1 hippocampal neurons under control and 14 days SMg conditions, respectively. **(C,F)** Magnified representative single neurons from **(B,E)** at 1000× magnification. Histogram in the lower panel shows percentage changes in mean (±SEM) perimeter and area of CA1 neurons in Nissl and Golgi–Cox-stained sections upon exposure to 14 days SMg condition as compared with control taken as 100%. ***P ≤ 0.001. Abbreviations: as in the text.

The mean (±SEM) perimeter [*F*(1, 7) = 39.89, *P* < 0.001] and area [*F*(1, 7) = 42.04, *P* < 0.001] of 14 days SMg-treated CA1 neurons decreased significantly compared with control (Figure 2; Table 1). However, the mean FF (±SEM) [*F*(1, 7) = 0.50, *P* = 0.506] values in treated rats were comparable to that of the control values (Table 1).

**Table 1:** **Nissl-stained CA1 neurons were analyzed after 14 days SMg and control conditions**.

Parameter of evaluation	Controls	14 Days SMg
	*N* = 4, *n* = 850 ± 50	*N* = 4, *n* = 850 ± 50
Area, μm^2^ (mean ± SEM)	148.85 ± 3.63	119.40 ± 2.73
		***P < 0.001
Perimeter, μm (mean ± SEM)	54.11 ± 0.61	48.75 ± 0.59
		***P < 0.001
FF factor	0.64 ± 0.01	0.63 ± 0.01
		*P* = 0.51

**N* = number of experiments; *n* = number of neurons studied*.

***P < 0.001 significantly different from control. Abbreviations: as in text.

### Golgi–Cox staining

Golgi-stained traced neurons of control and 14 days SMg-treated rats were analyzed using Neurolucida Explorer. It was found that there were significant differences in various parameters as given below.

#### Soma

**Figure 3:**
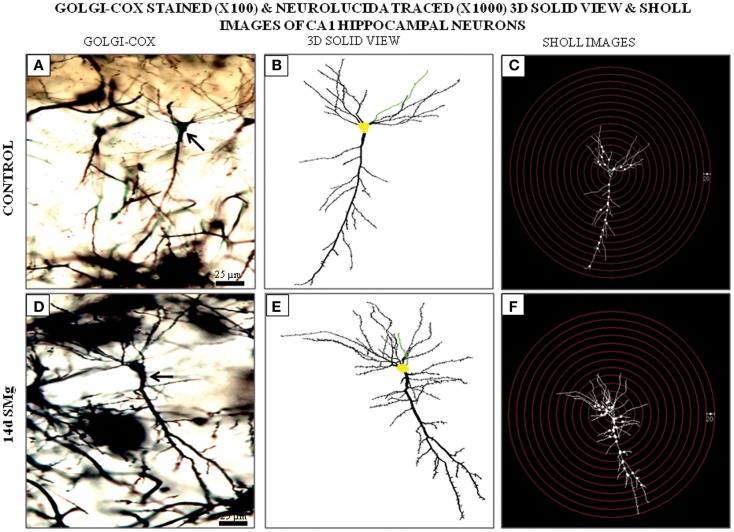
**Photomicrographs of Golgi–Cox-stained representative CA1 neurons (100×) from control (A) and 14 days SMg-exposed (D) rats are shown (arrow). (B,E) 3-D-traced neurons (1000×**) in area marked on **(A,D)**, respectively, using Neurolucida 9 and Neurolucida explorer 9. **(C,F)** Sholl images of traced neurons in **(B,E)** respectively, having concentric radii of 20 μm. Fourteen days SMg showed more branching as compared with control. Abbreviations: as in the text.

The mean (±SEM) perimeter [*F*(1, 11) = 20.60, *P* < 0.001] and area [*F*(1, 11) = 46.33, *P* < 0.001] of Golgi-stained 14 days SMg-treated CA1 neurons decreased significantly compared with that of the controls (Figures 2 and 3; Table 2); however, their FF values [*F*(1, 11) = 0, *P* = 1] were comparable to the controls.

**Table 2:** **Golgi–Cox-stained CA1 neurons were analyzed after 14 days SMg and control conditions**.

Parameter of evaluation	Controls (mean ± SEM)	14 Days SMg (mean ± SEM)
	*N* = 6, *n* = 28 ± 2	*N* = 6, *n* = 28 ± 2
Perimeter (μm)	59.44 ± 1.24	52.18 ± 1.01
		***P < 0.001
Area (μm^2^)	214.61 ± 6.16	165.13 ± 3.86
		***P < 0.001
FF factors	0.77 ± 0.02	0.77 ± 0.02
		*P* = 1
Nodes dendrite (D)	10.35 ± 0.60	12.68 ± 0.69
		*P = 0.029
Nodes apical dendrite (AD)	13.46 ± 0.56	16.48 ± 0.88
		*P = 0.016
Spines (D)	206.77 ± 13.62	283.96 ± 17.87
		**P = 0.006
Spines (AD)	178.96 ± 9.31	218.92 ± 17.19
		*P* = 0.068
Total length (D) (μm)	1099.91 ± 60.61	1289.08 ± 88.08
		*P* = 0.107
Total length (AD) (μm)	1301 ± 45.50	1424.24 ± 81.94
		*P* = 0.219
Spine density (D)	0.19 ± 0.007	0.24 ± 0.01
Spine per μm		**P = 0.002
Spine density (AD)	0.14 ± 0.007	0.16 ± 0.01
		*P* = 0.132
Nodes (D + AD)	23.81 ± 0.85	29.16 ± 0.92
		**P = 0.002
Spines (D + AD)	385.73 ± 19.03	502.88 ± 26.81
		**P = 0.005
Total length (D + AD) (μm)	2400.95 ± 73.61	2713.31 ± 137.06
		*P* = 0.072
Spine density (D + AD)	0.16 ± 0.006	0.19 ± 0.01
		*P = 0.028

**N* = number of experiments; *n* = number of neurons studied*.

***P ≤ 0.001 or **P ≤ 0. 01 or *P ≤ 0.05 significantly different from control. 14 days SMg, 14 days simulated microgravity; AD, apical dendrites; D, basal dendrites.

#### Basal dendrites

**Figure 4:**
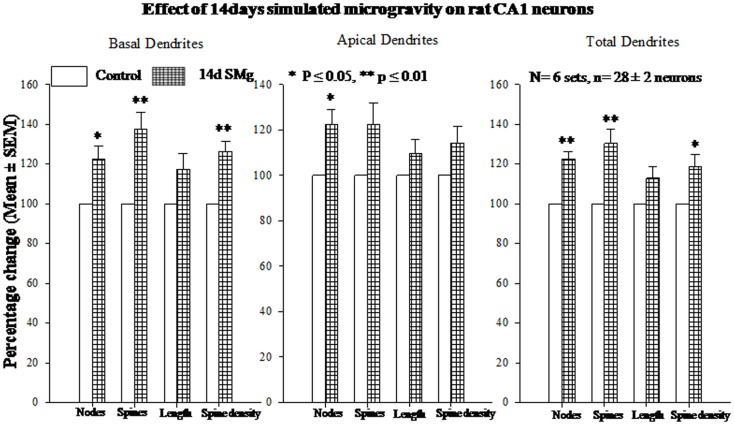
**Histograms showing percentage changes in mean (±SEM) number of nodes, spines, dendritic length, and spine density of traced Golgi–Cox-stained CA1 neurons upon exposure to 14 days SMg condition as compared with its control taken as 100% are represented by bars**. **P ≤ 0.01; *P ≤ 0.05 significantly different from control. *N* = total number of sets, *n* = total number of neurons studied. Abbreviations: as in text.

**Figure 5:**
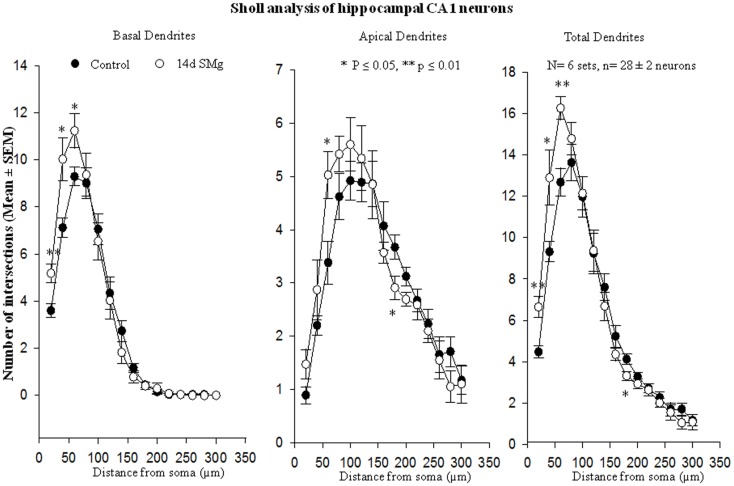
**Sholl analysis enabled us in evaluating basal, apical, total (basal+apical) dendrites of hippocampal CA1 neurons under SMg and control conditions**. The relative changes in various dendrites are represented in this figure. **P ≤ 0.01; *P ≤ 0.05 significantly different from control. *N* = total number of sets, *n* = total number of neurons studied. Abbreviations: as in text.

The mean (±SEM) of total number of nodes [*F*(1, 11) = 6.49, *P* = 0.029], spines [*F*(1, 11) = 11.80, *P* = 0.006], and spine density (spine/micrometer) [*F*(1, 11) = 16.78, *P* = 0.002] of 14 days SMg-exposed rats’ basal dendrites increased significantly compared with that of respective control values (Figures 3 and 4; Table 2). Sholl analysis also showed more branching of basal dendrites in rats exposed to SMg (Figure 5). However, the mean basal dendritic length [*F*(1, 11) = 3.13, *P* = 0.107] of neurons of 14 days SMg-exposed rats were comparable to that of respective controls (Figure 4; Table 2).

#### Apical dendrites

The mean (±SEM) number of nodes [*F*(1, 11) = 8.38, *P* = 0.016] of apical dendrites increased significantly after exposure to microgravity; while the mean (±SEM) number of spines [*F*(1, 11) = 4.178, *P* = 0.068], spine density [*F*(1, 11) = 2.685, *P* = 0.132], and apical dendritic length [*F*(1, 11) = 1.729, *P* = 0.218] were comparable to respective controls (Figures 3 and 4; Table 2).

#### Total (apical+basal) dendrites

Total dendrites showed similar pattern as that observed for basal dendrites. The mean (±SEM) number of nodes [*F*(1, 11) = 18.24, *P* = 0.002] and spines [*F*(1, 11) = 12.69, *P* = 0.005] as well as spine density [*F*(1, 11) = 6.618, *P* = 0.028] of total dendrites increased significantly after exposure to 14 days SMg (Figures 3 and 4; Table 2). However, the mean total dendritic length [*F*(1, 11) = 4.03, *P* = 0.072] of 14 days SMg-treated neurons was comparable to that of respective controls (Figure 4; Table 2).

### Transmission electron microscopy

**Figure 6:**
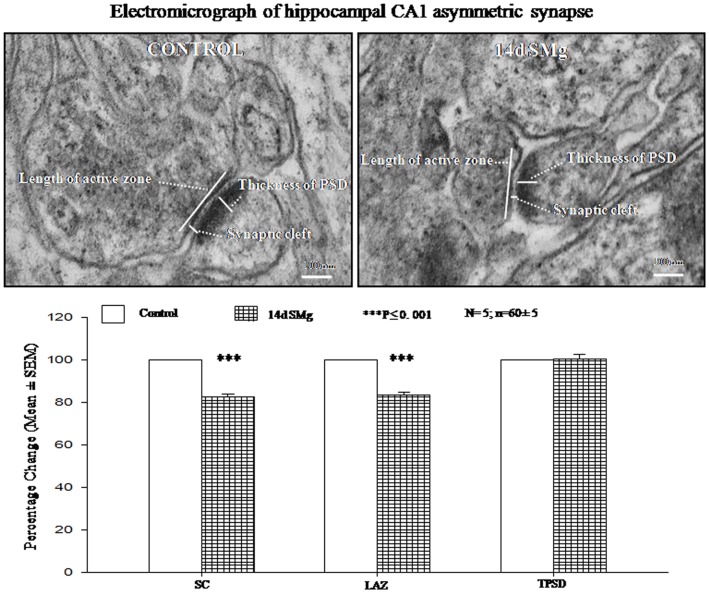
**Electron micrograph showing the ultrastructural features of synapses in CA1 area**. The synaptic cleft, length of active zone, and thickness of PSD are shown in the figure. Lower panel histogram shows percentage changes in mean (±SEM) SC, LAZ, and TPSD of CA1 neurons upon exposure of rats to 14 days SMg condition as compared with control taken as 100%. *N* = total number of sets, *n* = total number of synapses studied. Abbreviations: as in the text.

There were significant decrease in SC [*F*(1, 9) = 143.19, *P* < 0.001] and LAZ [*F*(1, 9) = 35.05, *P* < 0.001] of CA1 asymmetric synapses of 14 days SMg-treated rats compared with respective control values; however thickness of PSD [*F*(1, 9) = 0.025, *P* = 0.88] was comparable to that of its control values (Figure 6; Table 3).

**Table 3:** **Synapses were selected according to the criteria of Gray’s type-1 synapse**.

Parameter of evaluation	Controls	14 Days SMg
	(*N* = 5, *n* = 60 ± 5)	(*N* = 5, *n* = 60 ± 5)
SC (mean ± SEM) (nm)	25.41 ± 0.14	21.01 ± 0.34
		***P < 0.001
LAZ (mean ± SEM) (nm)	267.82 ± 6.06	223.52 ± 4.39
		***P < 0.001
Thickness of PSD (mean ± SEM) (nm)	42.64 ± 0.86	42.84 ± 0.92
		*P* = 0.88

*Selected CA1 synapses were analyzed after 14 days SMg and control conditions. *N* = number of experiments; *n* = number of synapses studied*.

***P < 0.001 significantly different from control. Abbreviations: as in text.

## Discussion

In the present study, we observed that as compared with controls the CA1 neurons of the rats exposed to 14 days SMg showed decreased area, perimeter, SC, and LAZ; however, the number of nodes, spines, and spine density were increased. The length of dendrites, FF, and thickness of PSD were comparable to control. Thus, our results showed significant changes in CA1 neuronal cytomorphometry and their connectivity when rats were exposed to SMg.

We observed that upon exposure to SMg although in absolute terms the soma size (perimeter, area, and FF) of the Nissl-stained neurons were smaller compared with that of changes observed in Golgi–Cox-stained neurons, their relative percentage change (the control value taken as 100%) was comparable (Figure 2). This is possibly because in Nissl stain molecules in the cytoplasm are stained, while in Golgi–Cox-stained neurons, the boundaries of the neurons are stained (32). Upon exposure to SMg, the soma FF values of CA1 neurons remained unaltered, which suggests that the decrease in area was proportional to the decrease in square of the perimeter of the cells as reported earlier (26). Such is possible in case of neurons having smooth surface and regular shape, i.e., without possessing significant number of somatic protrusions, appendages, or spines. This view may be supported by the fact that in our Golgi–Cox-stained sections, we observed that the cell bodies of the CA1 neurons possess very few somatic protrusions, appendages, and spines. Thus, our findings suggest that hippocampal CA1 neurons are more vulnerable, as reflected by alterations in their sizes, upon exposure to SMg than that of other rough surfaced neurons (26).

The active zones in the presynaptic terminals possess various types of SNARE (soluble *N*-ethyl-maleimide-sensitive factor attachment protein receptor) proteins (33), which mediate synaptic vesicle fusion with the presynaptic terminal membrane and exocytosis of neurotransmitters (34). Thus, our finding that SMg exposure induced down regulation of SNARE proteins may be interpreted as there would be reduced release of neurotransmitters in the rat brain after SMg exposure (35), which then would affect brain functions including behavior. Microgravity exposure-associated down regulation of many SNARE proteins in hippocampal neurons as has been reported earlier (2, 36) and reduced LAZ as observed in this study support our interpretation. Relative reduction of activation of CA1 neurons would cause reduced influx and reduced concentration of Na^+^ inside the neurons (37) resulting in exosmosis (38) and reduction in the size of CA1 neurons after exposure to 14 days SMg as discussed above.

Gravitational force acts in a particular plane of orientation and defines the spatial environment, which however, changes upon exposure to microgravity. Under our experimental SMg condition, afferent flow from the hind-limbs decreases due to unloading. In order to compensate for the lack of afferent flow, hippocampal neurons may respond with the creation of new synapses and re-arrangements of neuronal circuits (1). Thus, it is possible that the cytomorphometric changes, i.e., increase in number of nodes, spine, and spine density could be compensatory effects caused by SMg. However, basal and apical dendrites did not show similar responses; basal dendrites showed significant increase in number of nodes, spine, and spine density, while apical dendrites were comparable to respective controls. Previous study showed differential propensity for long-term potentiation (LTP) at basal and apical dendritic synapse of CA1 neurons (39). They concluded that, “*basal dendritic potentiation would increase and decrease relatively quickly, while the apical dendritic LTP would occur only after a certain degree of disinhibition*.” Thus, in our study, the basal dendrites were modulated differentially than that of apical dendrites after 14 days SMg, which is likely to have bearing with information processing by the neurons. We cannot comment on whether these changes are specific to hippocampal CA1 neurons, or are generalized phenomena, which need further study.

Visualization of dendritic arborization has bearing on staining methods like intracellular Nissl-staining or Golgi staining of neurons. The latter staining appears to show decreased dendritic visualization as compared with intracellular staining of hippocampal CA1 neurons and our findings on dendritic length are consistent with the results of Golgi-stained hippocampal CA1 neurons as reported earlier (40–49). Although classical Golgi method has a number of challenges for quantitative evaluation of neuronal morphology, it has the merit of providing a broad overview of the neuronal characteristics for comparative analysis. Therefore, our results may not be comparable with intracellular staining but they are sufficient for relative understanding of neuronal morphology. Further, as it is not possible that all neurons are evenly cut during sectioning, we have considered only those Nissl-stained selected neurons where nuclear boundaries were clearly visible. We were also aware that cytoplasmic staining does not allow a critical outline of cytoplasmic membrane. Hence, to minimize sampling error, we estimated a large number of randomly selected neurons (850 ± 50) across the hippocampal CA1 area (Figure 2) from four rats each of control and experimental groups. These measures served to significantly overcome the limitations associated to Nissl-staining while analyzing area and perimeter of neurons. As the intra-group data were comparable, the values were pooled for comparative analysis between control and experimental groups and determining the statistical significance.

Gravity influences most physiological processes (50). During space travel, astronauts are exposed to low gravity, which could affect neuronal cytomorphology resulting in changes in normal brain functions. Since there are significant limitations in conducting studies in space, including that of cost and opportunities, earth-based simulation studies have been designed to investigate the physiological responses to weightlessness. For example, clinostats for plants and non-motile cells (51), head-down bed rest for humans (52), water immersion for humans (53), HLS model for rodents (22, 23) have been used. Despite some shortcomings, in the absence of availability of better model for such studies, rodent HLS model has been widely accepted to simulate microgravity condition comparable to induced during space flight and to study its effect on physiological processes occurring due to unloading (22).

Hippocampal neurons are highly sensitive to stress (54) and atrophy of the thymus, adrenal hypertrophy, and corticosterone levels are indicators of stress. Accordingly, based on changes in these and additional parameters, the National Aeronautics and Space Administration (NASA) Ames Research Centre (ARC) Animal Care has approved HLS rat model for such studies. Although there were some disagreements on HLS-associated changes in adrenal hypertrophy (55) and corticosterone level (56–58) up to 7 day exposure to SMg, these parameters were comparable to normal on longer exposure; hence, we used longer (14 days exposure to SMg) paradigm as reported earlier (22). In our experiment during initial exposure to HLS, we observed fluctuations in body weight, stabilized by the 14th day, which was possibly an adaptive response to the new environment. The results were specific to SMg because control rats maintained in normal cages in the same room were unaffected. Notwithstanding, although our findings are suggestive, further studies are needed to understand the effects of chronic exposure to SMg.

Mammalian central nervous system has evolved within earth’s 1-g gravitational field and exposure to altered gravity may lead to cognitive and learning deficits (6, 8, 9). Exposure to SMg has been reported to impair learning and memory in rodents (8, 17), which is generally consistent with previous studies and space flight data (6, 59, 60). Relatively very few studies have investigated the effects of exposure to altered gravity on cognitive functions and whatever little are available, the results are inconclusive (61, 62); also, we have very little knowledge about their mechanism of action. It has been reported that initial 3 weeks in space mission and first 2 weeks after returning from prolonged space flight were critical for changes in learning and memory (63). In our case, 14 days exposure to SMg resulted in morphological differences in the CA1 neurons of rats, which could be likely indication of early cytomorphological changes for the induction and expression of future behavioral changes. The microgravity exposure-associated impairment of cognitive functions are likely to be dependent on the duration of exposure and the effects varied depending on pre-disposition, rate of recovery, and degree of adaptation. Previous studies showed loss of dendritic spines after suffering from cerebral stroke (64, 65). Subject to confirmation, on a positive note, based on our findings of growth of dendritic spines after exposure of SMg, as a possible therapy, we put forward a testable proposition that stroke patients may be exposed to controlled-SMg for growth of nodes, spines, dendrites for better and faster recovery. Also, since we are fast moving toward space travel (mars, moon missions, etc.), health of the space crew members is a major concern; our results may be useful to explore countermeasures and design effective prevention of the astronauts exposed to microgravity.

## Conclusion

We observed that hippocampal CA1 neurons of rats exposed to 14 days SMg although showed impaired cytomorphological characteristics and connectivity, their arborizations were increased. The latter could be a compensatory effect, which under specific conditions, e.g., for recovery in brain stroke patients, may be beneficial. Our results add to the limited knowledge available in the field and may be useful to explore countermeasures and to design effective prevention of symptoms especially in astronauts expressing deficits in cognitive functions; for which we lack detail study.

## Authors Contribution

Amit Ranjan collected and analyzed the data as well as participated in preparing this manuscript; Jitendra Behari extended help while planning the study. Birendra N. Mallick planned the study, arranged funds, and wrote the manuscript. All authors have read and approved the final manuscript.

## Conflict of Interest Statement

The authors declare that the research was conducted in the absence of any commercial or financial relationships that could be construed as a potential conflict of interest.
